# HYPERTRIGLYCERIDEMIC WAIST AND ASSOCIATED FACTORS IN CHILDREN AND ADOLESCENTS WITH TYPE 1 DIABETES MELLITUS

**DOI:** 10.1590/1984-0462/2020/38/2019073

**Published:** 2020-03-16

**Authors:** Lílian Caroline de Souza e Silva, Skalyt Lee Barbosa e Silva, Ávilla Monalisa Silva de Oliveira, Jacqueline Rosangela de Araujo, Ilma Kruze Grande de Arruda, Regiane Maio, Maria da Conceição Chaves de Lemos

**Affiliations:** a Universidade Federal de Pernambuco, Recife, PE, Brazil.

**Keywords:** Hypertriglyceridemic waist, Type 1 diabetes mellitus, Children, Cintura hipertrigliceridêmica, Diabetes mellitus tipo 1, Criança

## Abstract

**Objective:**

To assess the frequency of the hypertriglyceridemic waist phenotype and its associated factors in children and adolescents with type 1 diabetes mellitus.

**Methods:**

This is an observational analytical study with individuals with type 1 diabetes mellitus, aged 5 to 18 years, of both genders, followed in a university hospital in the Brazilian Northeast. Weight, height, and waist circumference were measured, and the lipid profile and glycated hemoglobin were analyzed. The hypertriglyceridemic waist phenotype was defined by the simultaneous presence of increased waist circumference (≥90^th^ percentile for age and gender) and elevated serum triglyceride levels (≥75 mg/dL for children and ≥90 mg/dL for adolescents). We also investigated the family history of cardiovascular diseases and diabetes, as well as sociodemographic and behavioral variables. In the statistical inference tests, the proportions were compared by Pearson’s chi-square test ­and/­or Fisher’s exact test, being significant p<0.05.

**Results:**

A total of 102 patients were evaluated, most of them females (54.9%) and adolescents (66.7%). The frequency of hypertriglyceridemic waist was 23.5%, which was associated with females (p=0.043), overweight (p=0.023), hypercholesterolemia (p=0.002), high LDL (p=0.001), and borderline VLDL (<0.001).

**Conclusions:**

The frequency of the hypertriglyceridemic waist phenotype was associated with females, atherogenic lipid profile, and overweight, indicating the importance of the nutritional monitoring of this population, aiming at reducing future cardiovascular diseases.

## RESUMO

**Objetivo:**

Avaliar a frequência do fenótipo cintura hipertrigliceridêmica e analisar seus fatores associados em crianças e adolescentes portadores de diabetes melito tipo 1.

**Métodos:**

Trata-se de um estudo observacional analítico com indivíduos com diabetes melito tipo 1, de cinco a 18 anos de idade, de ambos os sexos, acompanhados em um hospital universitário do Nordeste brasileiro. Foram realizadas medidas de peso, altura e circunferência da cintura, além da análise do perfil lipídico e da hemoglobina glicada. O fenótipo cintura hipertrigliceridêmica foi definido pela presença simultânea da circunferência da cintura aumentada (≥percentil 90 por idade e sexo) e dos níveis séricos de triglicerídeos elevados (≥75 mg/dL para crianças e ≥90 mg/dL para adolescentes). Investigaram-se, ainda, os antecedentes familiares para doenças cardiovasculares e diabetes, e também variáveis sociodemográficas e comportamentais. Nos testes de inferência estatística, as proporções foram comparadas pelo teste do qui-quadrado de Pearson e/ou exato de Fisher, sendo significante p<0,05.

**Resultados:**

Foram avaliados 102 pacientes, com predomínio do sexo feminino (54,9%) e de adolescentes (66,7%). A frequência de cintura hipertrigliceridêmica foi de 23,5%; a qual apresentou associação com o sexo feminino (p=0,043), excesso de peso (p=0,023), hipercolesterolemia (p=0,002), LDL elevado (p=0,001) e VLDL em valores limítrofes (<0,001).

**Conclusões:**

A frequência do fenótipo cintura hipertrigliceridêmica foi associada ao sexo feminino, ao perfil lipídico aterogênico e ao excesso ponderal, evidenciando a importância do acompanhamento nutricional dessa população, visando à redução de agravos cardiovasculares futuros.

## INTRODUCTION

Type 1 diabetes mellitus (T1DM) consists of a heterogeneous group of metabolic disorders resulting from the partial or total destruction of beta cells from pancreatic islets of Langerhans, leading to the progressive inability to produce insulin and, subsequently, hyperglycemia.^1^ Estimates indicate that 5 to 10% of Brazilians with diabetes mellitus (DM) have T1DM, affecting mainly children and adolescents, since this condition is often diagnosed during childhood.^2^

Due to the process of nutritional transition and profile modification in the world population in recent decades, the nutritional status of children and adolescents with T1DM has been similar to that of healthy individuals, with evidence of high prevalence of overweight and obesity in this group.^3^ Changes in lifestyle contribute to excess weight due to the lower dietary restriction provided by flexible insulin therapy regimen,^4^ as well as the reduced energy expenditure, favored by the longer time spent in front of electronic devices, such as television, video games, among others.^5^

Considering that overweight is a major risk factor for metabolic disorders and cardiovascular diseases (CVD), patients with T1DM require further follow-up. These individuals also present an early onset of severe atherosclerosis when compared to the healthy population of the same age group due to vascular damage caused by hyperglycemia.^6^ Therefore, sensitive clinical and/or laboratory methods for identifying risk factors should be part of the outpatient care routine in this group.

An index recently used to predict these disorders is the hypertriglyceridemic waist (HTGW) phenotype, defined by the simultaneous presence of increased waist circumference (WC) and elevated serum triglyceride levels.^7^ This tool has been proposed as an alternative to the diagnosis of metabolic syndrome, standing out as a marker of cardiovascular and metabolic risk and its association with visceral obesity,^8^ as it includes WC, an anthropometric measure related to central obesity and hypertriglyceridemia, and predictors of metabolic syndrome ­and/­or atherogenic metabolic triad - hyperinsulinemia and high levels of apolipoprotein B and low-density lipoprotein (LDL cholesterol).^9^

In 2000, Lemieux et al. developed one of the first studies involving HTGW with a sample of Canadian male adults, finding high agreement between this index and the atherogenic metabolic triad.^10^ Nevertheless, despite the lack of studies involving individuals with T1DM nationwide, the literature has presented research with several populations, including children^11^ and adolescents,^8^ ^,^ ^12^ indicating a scenario in which CVDs represent the main cause of morbidity and mortality, affecting increasingly younger individuals and significantly reducing productive life. Thus, this study aimed to evaluate the frequency of the HTGW phenotype and its associated factors in children and adolescents with T1DM.

## METHOD

This is an observational analytical study conducted at a university hospital in Recife, Pernambuco, Northeastern Brazil. The population consisted of children and adolescents with T1DM, of both genders, aged 5 to 18 years, followed in an outpatient clinic.

This investigation used a convenience sample and evaluated all children and adolescents with T1DM from April to September 2018 who met the following inclusion criteria: age between 5 and 18 years and recent biochemical tests (prior three months). We excluded individuals whose characteristics could influence anthropometric measurements and other variables, such as cerebral palsy, Down syndrome, or any other genetic changes; patients recently submitted to abdominal surgery; carriers of inborn errors of metabolism (celiac disease, galactosemia, cow’s milk protein allergy); those with chronic kidney disease or on corticosteroid therapy.

The data collected included gender and age, as well as level of schooling and monthly income of parents and/or guardians. The level of schooling was categorized into years of study, as follows: less than eight years and eight or more years of study. Similarly, family income was divided into: up to one minimum wage (R$ 954.00) and more than one minimum wage (>R$ 954.00).

The anthropometric evaluation involved measurements of weight, height, and WC, calculated twice, according to Lohman’s criteria.^13^ Weight was measured on a Filizola mechanical scale (São Paulo, Brazil) and height on the stadiometer attached to the same scale. The nutritional diagnosis was determined according to the body mass index (BMI) curves for age, recommended by the World Health Organization in 2007 for children and adolescents aged 5 to 19 years, and classified based on Z-scores.^14^ WC was measured using a non-elastic measuring tape placed at the midpoint between the iliac crest and the inferior margin of the last rib. The cut-off point adopted for increased WC was the ≥90^th^ percentile for age and gender.^15^ A resident nutritionist of the hospital responsible for the study performed all anthropometric measurements.

The biochemical variables used were: total cholesterol (TC), high-density lipoprotein (HDL cholesterol), LDL cholesterol, very low-density lipoprotein (VLDL cholesterol), and triglycerides (TG), analyzed by automation, and glycated hemoglobin (HbA1c), analyzed by immunoturbidimetry. To this end, we consulted the results of tests performed at the clinical analysis laboratory of the Hospital das Clínicas of the Universidade Federal de Pernambuco (UFPE) in up to three months. We adopted HbA1c <7.5% as a reference value to evaluate glycemic control;^1^ while the lipid profile had the following reference values: TC <170 mg/dL; HDL cholesterol >45 mg/dL; LDL cholesterol <110 mg/dL; VLDL: desirable <30 mg/dL and borderline between 30 and 67 mg/dL; TG <75 mg/dL (0 to 9 years old) and <90 mg/dL (10 to 19 years old), following the Brazilian Guideline for Dyslipidemia and Atherosclerosis Prevention.^16^

The HTGW phenotype was defined in children and adolescents as increased WC (≥90^th^ percentile for age and gender) associated with hypertriglyceridemia (≥75 mg/dL for children and ≥90 mg/dL for adolescents).^7^

The behavioral variables investigated the practice of physical activity and screen time. The first one questioned whether or not the participant practiced physical activity and, if so, to inform which modalities, as well as the duration and weekly frequency. In turn, screen time represents a sedentary behavior and corresponds to the time spent using electronic devices. The daily use of these devices was dichotomized into less than two hours and two or more hours per day, following the American Academy of Pediatrics, which recommends two hours as the maximum screen time for children over two years and adolescents.^5^

We also evaluated the time since diagnosis of T1DM and the start of treatment, as well as family history for CVD and DM. Positive family history of CVD was defined as the patient having at least one close relative (parents, siblings, or grandparents) with an episode of arterial hypertension, coronary artery disease, heart failure, cerebrovascular accident, and/or peripheral vascular disease; and of diabetes, as a close relative having type 1 or 2 DM.

For statistical analysis, data were entered into the software Microsoft Office Excel and analyzed in the Statistical Package for the Social Sciences (SPSS) version 13.0 (SPSS Inc., Chicago, IL, USA) and Epi-Info version 3.5.4 (CDC, Atlanta, GA, USA). Continuous variables were tested for normality by the Kolmogorov-Smirnov test; those with normal distribution were expressed as mean and standard deviation, and the ones with non-normal distribution, as median and interquartile range (IQR). The description of proportions approximated the binomial distribution to the normal distribution with the 95% confidence interval (95%CI). In the statistical inference tests, the proportions were compared by Pearson’s chi-square test and/or Fisher’s exact test. We adopted a 5.0% significance level to reject the null hypothesis.

This project is part of a study called “Hypertriglyceridemic Waist and Cardiovascular Risk in Children and Adolescents,” approved by the Human Research Ethics Committee of UFPE, complying with Resolution No. 466/2012 of the National Health Council, under CAAE No. 83335318.2.0000.5208. Data collection started after the legal guardian signed the informed consent form (ICF), authorizing the participation of the minor in the research, and the subjects signed the agreement form (AF), stating his or her free and voluntary decision to participate in the study.

## RESULTS

The final sample consisted of 102 patients. However, since these data were collected from medical records, HbA1c could be evaluated in only 99 patients, TC in 100, LDL and HDL fractions in 98, and VLDL in 74.

We found a prevalence of females (54.9%) and adolescents (66.7%). Most participants lived in the inland of the state of Pernambuco (49%) and had a family income lower than one minimum wage (68.6%), and 62.7% of the parents or guardians had eight years or more of schooling (Table 1).

**Table 1:** Socioeconomic, demographic, and clinical characteristics of children and adolescents with type 1 diabetes mellitus, Hospital das Clínicas, Universidade Federal de Pernambuco, 2018.

Variables	n	%	95%CI
Age			
Children (5 to 10 years)	34	33.3	24.3-43.4
Adolescents (11 to 19 years)	68	66.7	56.6-75.7
Gender			
Female	56	54.9	44.7-64.8
Origin			
Recife	22	21.6	14.0-30.8
Metropolitan area	30	29.4	20.8-39.3
Inland	50	49.0	39.0-59.1
Schooling of the main caregiver			
<8 years	38	37.3	27.9-47.4
Family income			
≤1 minimum wage	70	68.6	58.7-77.5
Family history			
Cardiovascular disease	77	75.5	66.0-83.5
Diabetes mellitus	75	73.5	63.9-81.8

95%CI: 95% confidence interval.

Regarding clinical variables, we identified positive family history of CVD and DM in 75.5 and 73.5%, respectively (Table 1). The median time to diagnosis and the beginning of treatment was 48 months (IQR: 24-84) in both.

Although 70.6% reported screen time of two hours or more per day (Table 2), 52% declared practicing physical activity, with an average weekly frequency of 3.6 times/week (±1.6) and a median of 60 minutes (IQR: 60-90) per day.

**Table 2:** Anthropometric and behavioral characteristics of children and adolescents with type 1 diabetes mellitus, Hospital das Clínicas, Universidade Federal de Pernambuco, 2018.

Variables	n	%	95%CI
Physical activity practice	53	52.0	41.8-62.0
Type of physical activity			
Dance	4	7.5	2.1-7.9
Cycling	17	32.1	19.5-45.6
Soccer	16	30.2	18.0-43.6
Walking	9	17.0	7.9-29.3
Other (Pilates, weight training, etc.)	7	13.2	6.6-27.1
Screen time			
≥2 hours	72	70.6	60.7-79.2
Body mass index/age			
Underweight	1	1.0	0.0-5.3
Normal	71	69.6	59.7-78.3
Overweight	26	25.5	17.4-35.1
Obesity	4	3.9	1.1-9.7
Increased waist circumference	46	45.1	35.2-55.3
Hypertriglyceridemic waist	24	23.5	15.7-33.0

95%CI: 95% confidence interval; body mass index/age: underweight (Z-score<-2), normal (Z-score between -2 and 1), overweight (Z-score between 1 and 2), obesity (Z-score>2); waist circumference: normal <90^th^ percentile, increased ≥90^th^ percentile.

With respect to the anthropometric assessment, 29.4% of participants were overweight (BMI/age with Z-score ≥1). A total of 45.1% of the population presented WC above the 90^th^ percentile, of which 23.5% had hypertriglyceridemia, thus characterizing the HTGW phenotype (Table 2).

The lipid profile evidenced hypercholesterolemia in 44% of the sample, while high LDL, reduced HDL, and borderline VLDL were identified in 24.5, 25.5, and 10.8%, respectively (Table 3). The mean HbA1c was 9.4% (±1.8) and was high in 91.9% of the individuals assessed.

**Table 3:** Metabolic profile of children and adolescents with type 1 diabetes mellitus, Hospital das Clínicas, Universidade Federal de Pernambuco, 2018.

Variables	n	%	95%CI
Increased total cholesterol	44	44.0	34.1-54.3
Increased LDL	24	24.5	16.4-34.2
Reduced HDL	25	25.5	17.2-35.3
Borderline VLDL	8	10.8	4.8-0.2
Increased triglycerides	39	38.2	28.8-48.4
Increased glycated hemoglobin	91	91.9	84.7-96.4

95%CI: 95% confidence interval; total cholesterol: normal <170 mg/dL, increased ≥170 mg/dL; LDL: low-density lipoprotein: normal <110 ­mg/­dL, increased ≥110 mg/dL; HDL: high-density lipoprotein: normal ≥45 ­mg/­dL, reduced <45 mg/dL; VLDL: very low-density lipoprotein: desirable <30 mg/dL, borderline 30 to 60 mg/dL; triglycerides: normal up to 9 years <75 mg/dL, 10 to 19 years <90 mg/dL, increased up to 9 years ≥75 mg/dL, 10 to 19 years ≥90 mg/dL; glycated hemoglobin: adequate <7.5%, increased ≥7.5%.

The factors associated with HTGW were the female gender (p=0.043), overweight (p=0.023), hypercholesterolemia (p=0.002), high LDL (p=0.001), and borderline VLDL (p<0.001). In addition, we detected a trend toward reduced HDL among patients with HTGW (p=0.076). As for the remaining variables, including age, we found no statistical association (Table 4).

**Table 4:** Association of hypertriglyceridemic waist with demographic, clinical, behavioral, anthropometric, and biochemical characteristics of children and adolescents with type 1 diabetes mellitus, Hospital das Clínicas, Universidade Federal de Pernambuco, 2018.

	Hypertriglyceridemic waist		
	n	%	p-value
Age			
Children	6	17.6	0.458^a^
Adolescents	18	26.5	
Gender			
Female	18	32.1	0.043^a^
Male	6	13.0	
Family history of cardiovascular disease			
Yes	20	26.0	0.453^a^
No	4	16.0	
Family history of diabetes mellitus			
Yes	20	26.7	0.327^a^
No	4	14.8	
Physical activity			
Yes	10	18.9	0.357^a^
No	14	28.6	
Screen time			
<2 hours	6	20.0	0.775^a^
≥2 hours	18	25.0	
Total cholesterol			
Normal	6	10.7	0.002^a^
Increased	17	38.6	
Low-density lipoprotein (LDL)			
Normal	10	13.5	0.001^a^
Increased	12	50.0	
High-density lipoprotein (HDL)			
Normal	12	16.4	0.076^a^
Reduced	9	36.0	
Very low-density lipoprotein (VLDL)			
Desirable	12	18.2	<0.001^b^
Borderline	7	87.5	
Glycated hemoglobin			
Adequate	2	25.0	1.000^b^
Increased	20	22.0	
Body mass index/age			
Underweight/normal	12	16.7	0.023^a^
Overweight/obesity	12	40.0	

^a^ Pearson’s chi-square test; ^b^ Fisher’s exact test; total cholesterol: normal <170 mg/dL, increased ≥170 mg/dL; LDL: normal <110 ­mg/­dL, increased ≥110 mg/dL; HDL: normal ≥45 mg/dL, reduced <45 ­mg/­dL; VLDL: desirable <30 mg/dL, borderline 30 to 60 mg/dL; glycated hemoglobin: adequate <7.5%, increased ≥7.5%; body mass index/age: underweight/normal (Z-score<1), overweight/obesity (Z-score≥1).

## DISCUSSION

The study of the HTGW phenotype, especially in children, has shown epidemiological relevance, since the atherosclerotic process begins in childhood and negatively influences the quality of life in adulthood, due to increased morbidity and mortality.^17^ ^,^ ^18^ The frequency of the phenotype in this investigation was 23.5%, showing that the population of children and adolescents with T1DM has followed the profile of non-carriers, with an increase in overweight, including in the abdominal region, which is probably a reflection of the nutritional transition process that has affected different age groups in both developed and developing countries.^19^

No research has evaluated HTGW in pediatric T1DM patients, and studies are limited to samples of healthy children and adolescents. Alavian et al.^17^ conducted an investigation in Iran with 5,625 students from a national school surveillance program (Caspian Study) and found a prevalence of 8.52%. In England, Bailey et al.^20^ identified that 7.3% of the students presented the phenotype and, therefore, higher cardiometabolic risk.

Nationwide, Guilherme et al.^21^ obtained results similar to those found in this study in research with students aged 10 to 14 years from public and private schools in Paraná, of whom 20.7% presented simultaneously increased WC and serum TG. On the other hand, lower percentages have also been documented in the scientific literature. In a cohort with students from a city in Bahia involving children and adolescents, Costa et al.^12^ found a prevalence of 10.6%. In studies conducted by Pereira et al.^11^ and Conceição-Machado et al.,^8^ the phenotype was prevalent only in adolescents, with 6.4 and 7.2%, respectively. These differences in prevalence can be explained by the different cut-off points adopted to classify WC and TG, and subsequent definition of the HTGW phenotype.

The BMI/age showed excess weight in 29.4% of the participants, of whom 25.5% were overweight and 3.9%, obese. In a multicenter population-based study, Liu et al.^3^ detected a prevalence of 22.1% of overweight and 12.6% of obesity among participants with T1DM aged 3 to 18 years in the United States. In Brazil, Marques et al.^4^ found lower proportions, with overweight in 14.1% of the sample, according to BMI/age curves.

However, globally, overweight has been increasing among individuals with T1DM over time, given that current studies report prevalence around 40%.^22^ ^,^ ^23^ Among the factors that have contributed to this scenario, the contemporary lifestyle stands out for its high energy consumption, including the elevated intake of dietary products, and maintenance of sedentary behaviors,^24^ reflected in this investigation as the excessive use of electronic devices by most of the sample (70.6% ), in addition to the lack of physical activity, also evidenced in large proportion (48%).

Another cause is the intensive treatment, characterized by the use of multiple doses of insulin, which, despite favoring glycemic control, tends to increase body weight and adiposity, particularly in the central region, due to its anabolic function.^25^

Regarding changes in lipid profile, Homma et al.^26^ reported higher data than those presented in this investigation, with hypercholesterolemia and elevated LDL in 56.7 and 44%, respectively. The proportion of volunteers with reduced HDL was similar to our sample, while hypertriglyceridemia was lower, affecting only 11.8%.

The prevalence of dyslipidemia has been higher among patients with the disease when compared to healthy children and adolescents. This is a major issue, since DM alone is considered a risk factor for early atherosclerosis among young individuals. Thus, this condition becomes an additional risk.^27^ Dyslipidemia secondary to inadequate glycemic control is a reality among individuals with T1DM, and high TC, LDL, and TG, as well as reduced HDL, have been substantially associated with elevated HbA1c.^26^

In this context, despite the adoption of intensive insulin regimens, adequate glycemic control is still a challenge for T1DM patients.^28^ In the cases evaluated, glycemic inadequacy, assessed via HbA1c, was present in approximately 90% of patients, increasing the atherogenic risk in this population and corroborating the results of other studies.^4^ ^,^ ^27^

Therefore, effective multidisciplinary care is necessary for this group, with more frequent medical appointments and educational actions for patients and their families. Nutritional monitoring is extremely relevant to achieve metabolic control, particularly knowing how to calculate the intake of carbohydrates, and the dietitian must customize the food schedule according to the patient’s daily routine.

 The factors associated with the HTGW phenotype include the female gender, suggesting a higher risk of chronic complications in girls, supporting the data from Homma et al.^26^ and Pérez et al.,^29^ which demonstrated that T1DM has a greater impact on increased cardiovascular risk among these adolescents, even those with adequate weight and glycemic control, indicating the female gender as an independent risk factor.

Another association evidenced was overweight, which may be justified by the fact that the higher the BMI, the greater the WC and, therefore, the serum triglyceride levels. Similar results were reported in a study with non-T1DM patients, who, in addition to obesity, showed a relationship between the phenotype and other cardiometabolic events, such as increased blood pressure, hypercholesterolemia, and reduced HDL.^30^

As for the lipid profile, HTGW was associated with hypercholesterolemia, elevated LDL, and borderline VLDL, which may be explained by the excessive lipolytic activity in adipose tissue located in the central region, resulting in an increase in circulating free fatty acids that work as a substrate for cholesterol and lipoprotein synthesis.^16^ Consequently, this association was expected. Despite the lack of association with HDL cholesterol, we found a trend toward reduced levels among those with HTGW, which deserves attention due to the protective role of this lipoprotein, especially against vascular damage.^16^

Besides, as hypertriglyceridemia results from excessive carbohydrate consumption, and VLDL is a triglyceride-rich lipoprotein responsible for its transport to peripheral tissues, the association between abnormal VLDL and HTGW may be justified by the predominant glycemic inadequacy in the sample, since this lipoprotein is carbohydrate-dependent. Similarly, Conceição-Machado et al.^21^ also evidenced a correlation between this phenotype and the atherogenic lipid profile in a healthy population of the same age group, recommending the use of this tool as a practical way of screening children and adolescents with cardiometabolic abnormalities.

This study has some limitations, such as the study design, which does not allow the establishment of causality, as it was carried out in a single moment. We also emphasize the lack of biochemical tests in some participants, preventing us from evaluating the metabolic profile of the sample in its entirety, as well as the absence of information regarding the total daily insulin dose and insulin regimen, limiting a better assessment of glycemic control. Lastly, the scarcity of studies involving the phenotype in patients with T1DM restricts the data that can be compared with the results presented in this investigation, reinforcing the need for further research on this subject in this public.

In conclusion, the frequency of the HTGW phenotype was similar and even higher than that of other studies carried out with healthy children and adolescents, being associated with the female gender, hypercholesterolemia, high levels of LDL and VLDL, and overweight. In this scenario, adopting preventive measures is necessary to reduce health problems, promote health, and, therefore, ensure quality of life.
